# Classification of Cirrhotic Patients with or without Minimal Hepatic Encephalopathy and Healthy Subjects Using Resting-State Attention-Related Network Analysis

**DOI:** 10.1371/journal.pone.0089684

**Published:** 2014-03-19

**Authors:** Hua-Jun Chen, Yu Wang, Xi-Qi Zhu, Pei-Cheng Li, Gao-Jun Teng

**Affiliations:** 1 Jiangsu Key Laboratory of Molecular and Functional Imaging, Department of Radiology, Zhongda Hospital, Medical School, Southeast University, Nanjing, China; 2 Department of Radiology, The Second Hospital of Nanjing, Medical School, Southeast University, Nanjing, China; Institute of Automation, Chinese Academy of Sciences, China

## Abstract

**Background:**

Attention deficit is an early and key characteristic of minimal hepatic encephalopathy (MHE) and has been used as indicator for MHE detection. The aim of this study is to classify the cirrhotic patients with or without MHE (NMHE) and healthy controls (HC) using the resting-state attention-related brain network analysis.

**Methods and Findings:**

Resting-state fMRI was administrated to 20 MHE patients, 21 NMHE patients, and 17 HCs. Three attention-related networks, including dorsal attention network (DAN), ventral attention network (VAN), and default mode network (DMN), were obtained by independent component analysis. One-way analysis of covariance was performed to determine the regions of interest (ROIs) showing significant functional connectivity (FC) change. With FC strength of ROIs as indicators, Linear Discriminant Analysis (LDA) was conducted to differentiate MHE from HC or NMHE. Across three groups, significant FC differences were found within DAN (left superior/inferior parietal lobule and right inferior parietal lobule), VAN (right superior parietal lobule), and DMN (bilateral posterior cingulate gyrus and precuneus, and left inferior parietal lobule). With FC strength of ROIs from three networks as indicators, LDA yielded 94.6% classification accuracy between MHE and HC (100% sensitivity and 88.2% specificity) and 85.4% classification accuracy between MHE and NMHE (90.0% sensitivity and 81.0% specificity).

**Conclusions:**

Our results suggest that the resting-state attention-related brain network analysis can be useful in classification of subjects with MHE, NMHE, and HC and may provide a new insight into MHE detection.

## Introduction

Minimal hepatic encephalopathy (MHE) (HE) is a common neurological complication of liver cirrhosis, which has a negative impact on the patients’ health-related quality of life [1] and is associated with bad outcomes [2]. Attention deficit is an early and key characteristic of MHE [3], [4], [5], [6], which is associated with patients’ falls and can result in impaired driving skills [7] and increased risk of motor vehicle accidents [8]. However, the neuropathological mechanisms underlying attention deficit in MHE are not completely understood so far, although it is reported that MHE patients show dysfunction in various attention subsystems [3], [4].

The resting-state fMRI studies have distinguished two attention networks by measuring spontaneous neuronal activity [9]: the dorsal attention network (DAN), which is responsible for the endogenous attention orienting (“top-down”) process, and the ventral attention network (VAN), which engages in the exogenous attention re-orienting (“bottom-up”) process. These two attention networks are involved in a model suggesting that different attentional operations during sensory orientation are carried out by interaction and cooperation between the two separate attention systems [10]. On the other hand, the spontaneous neuronal activity in the so-called default mode network (DMN), a resting-state network characterized by consistently task-induced deactivation, is anti-correlated with that in the attention networks [11]. It is believed that deactivation in the DMN is essential to reallocate neuronal resources toward behaviorally relevant processes [12], [13].

Notably, MHE is currently considered as a neurological disease with the disrupted functional connectivity (FC) in the attention-related loops [14], [15], [16], [17]. However, the existing studies do not investigate whether both endogenous and exogenous attention processes are simultaneously impaired in MHE and examine the feasibility of classifying patients with or without MHE and healthy controls (HC) using resting-state attention-related network analysis. In fact, previous studies have shown that MHE can be detected by measuring attention deficit in cirrhotic patients [5], [18]. Moreover, recent reports have demonstrated the utility of attention-related network analysis based on resting-state functional MR imaging in providing diagnostic biomarkers for neurological diseases with attention decline (e.g. Alzheimer disease [19]). In the context, we aimed to examine the changes of resting-state FC in attention-related networks in MHE patients and to investigate whether these alterations can be used to classify the cirrhotic patients with or without MHE and HC.

## Materials and Methods

### 2.1 Participants

This study was approved by the Research Ethics Committee of Affiliated Zhongda Hospital, Southeast University. Seventeen HC, 21 cirrhotic patients without (NMHE), and 20 cirrhotic patients with MHE were enrolled after providing written consent. The demographic and clinical data are summarized in Table 1. All subjects included were 40∼60 years old and their education years were 5∼11 years. MHE was diagnosed by the neuropsychiatric tests, including Number Connection Test A (NCT-A), Digit Symbol Test (DST), and Block Design Test (BDT). DST and BDT are subtests of the Wechsler Adult Intelligence Scale-Revised for China (WAIS-RC). These tests have been widely used to diagnose MHE [20], [21], [22], [23]. Patients were diagnosed as MHE, if they had abnormal score in any of the three neurocognitive tests [8], [16], [24], [25]. The normal values of the neurocognitive tests were determined from 160 healthy volunteers, who were age- and education-matched to the patients in this study. Normal values were expressed as mean ±2 standard deviations. If test score was more than 2 standard deviations beyond mean value, it was considered as abnormality [8], [16], [24], [25].

**Table 1:** Demographic and clinical characteristics of subjects.

Characteristic	Healthy Control	NMHE patients	MHE patients	p-value
	(*n* = 17)	(*n* = 21)	(*n* = 20)	(ANOVA)
Age (year)	49.7±5.1	50.1±5.7	52.0±5.2	0.36
Sex (Male/Female)	14/3	20/1	18/2	0.43 (χ^2^-test)
Education level (year)	8.2±2.2	8.7±2.0	8.1±2.0	0.61
Etiology of cirrhosis (HBV/alcoholism/HBV+alcoholism)	–	17/4/0	16/0/4	–
Child–Pugh stage (A/B/C)	–	14/5/2	5/6/9	–
Number Connection Test A (seconds)	48.2±15.2	44.9±13.2	77.1±15.3*	<0.001
Digit symbol test (raw score)	41.8±7.4	41.6±7.5	24.0±6.5*	<0.001
Block design test (raw score)	30.6±8.1	28.0±7.7	18.0±6.7*	<0.001

Note: HE, hepatic encephalopathy; NMHE, the cirrhotic patients without MHE; MHE, minimal hepatic encephalopathy; ANOVA, analysis of variance. * indicates that MHE patients had significantly worse neurological performances as compared to two other groups. There was no significant difference in neurological performance between healthy controls and NMHE patients.

Exclusion criteria included current HE or history of other neuropsychiatric diseases, severe organic diseases (such as cardiac disease, advanced pulmonary disorders, and renal failure), taking psychotropic medications, uncontrolled endocrine or metabolic diseases (such as diabetes mellitus and thyroid dysfunction), and alcohol abuse 6 months prior to the study.

### 2.2 MRI Data Acquisition

MRI data were collected using a 1.5 T scanner (Vantage Atlas; TOSHIBA). The participants were instructed to rest with their eyes closed, “not to think of anything in particular”, and keep their heads still during fMRI scanning. Functional images were collected with an echo planar imaging sequence (TR/TE = 2500 ms/40 ms, FOV = 24 cm × 24 cm, matrix = 64 × 64, FA = 90°, slice thickness/gap = 5 mm/1 mm, 22 axial slices) to measure 120 brain volumes. T1-weighted images were also obtained with the following parameters: 108 sagittal slices, FOV = 256 mm × 256 mm, matrix = 256 × 256, slice thickness/gap = 1.5 mm/0 mm.

### 2.3 Image Processing

**Figure 1:**
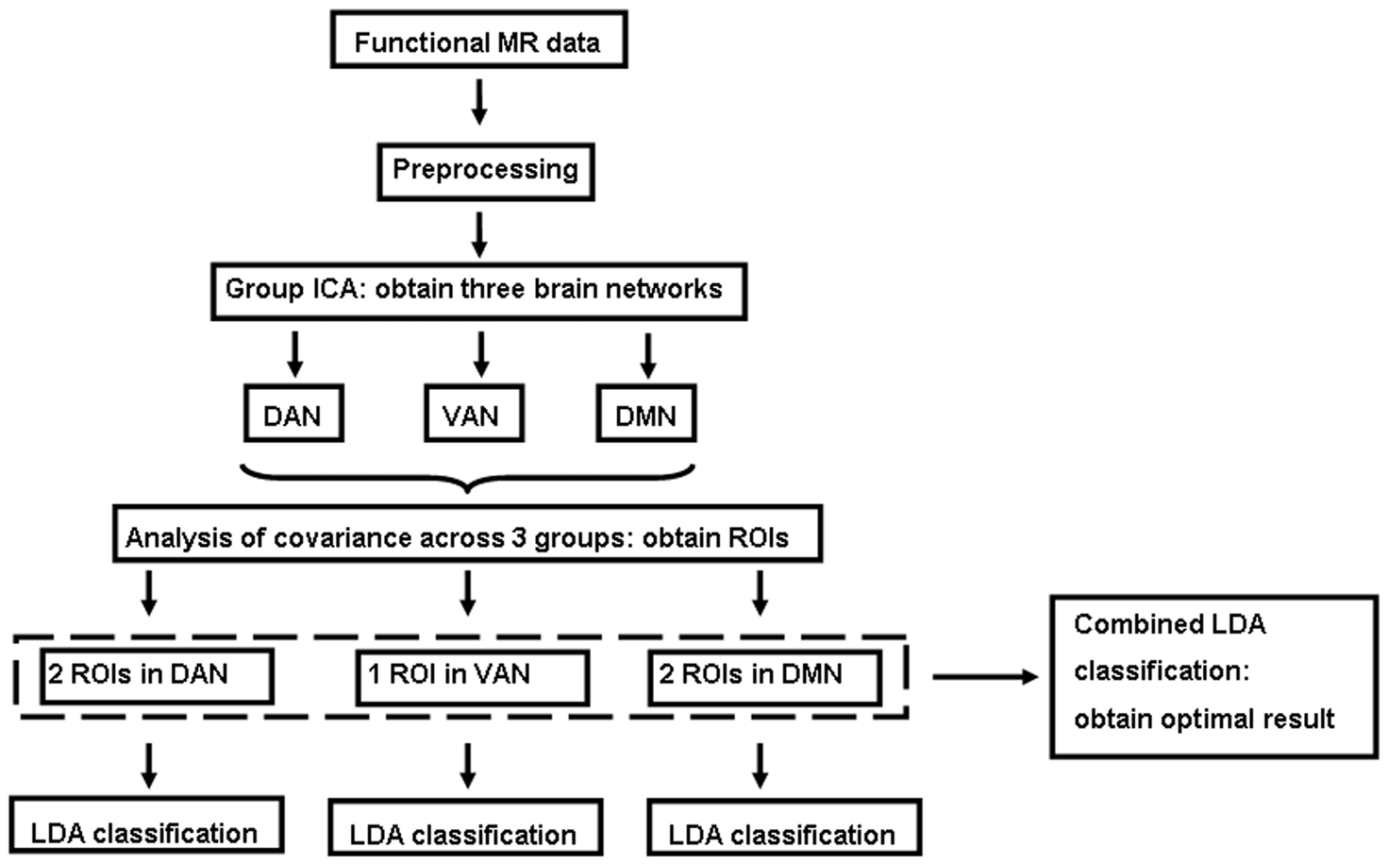
Summary of image processing and linear discriminant analysis. DAN, dorsal attention network; VAN, ventral attention network; DMN, default mode network; ROI, region of interest; LDA, linear discriminant analysis.

Image analysis was performed using Statistical Parametric Mapping (SPM5) software (http://www.fil.ion.ucl.ac.uk/spm), Data Processing Assistant for Resting-State fMRI (DPARSF) tool [26], and GIFT software (http://icatb.sourceforge.net). All image processing steps described below are summarized in Figure 1.

#### Functional data preprocessing

Functional imaging data were preprocessed using DPARSF tool [26]. The first 8 volumes of functional images were discarded to reach a steady-state magnetization and allow subjects to adapt to the scanning environment. The remaining consecutive volumes were used for slice-timing adjustment and realignment for head-motion correction. None of the subjects was found to have excessive head movement (translation >2.0 mm or rotation >2.0°). Subsequently, the functional images were spatially normalized to the Montreal Neurological Institute (MNI) space and were resampled into a voxel size of 3 × 3 × 3 mm^3^. Finally, functional images were smoothed with an isotropic Gaussian kernel (FWHW = 4 mm).

#### Independent component analysis (ICA)

Group ICA was conducted using GIFT software (http://icatb.sourceforge.net), which included data reduction by two rounds of principal component analysis (PCA), ICA separation and back-reconstruction [27]. In the first round of PCA, the data of each subject was temporally dimension-reduced to the 55 components. After concatenation across subjects within group, the dimensions were again reduced into 45 components via the second round of PCA. The spatial independent component analysis was conducted to decompose the data using the Infomax algorithm. For each IC, the waveform corresponds to the time course of a specific pattern of brain networks, and the intensity with which the connectivity is found across the voxels is expressed in the associated spatial map [28]. To display the voxels that contributed most strongly to a particular IC, the time courses and spatial maps for each subject were computed and converted into Z values. The Z values in the individual component map represented the fit (strength of FC) of a specific voxel time course to the group-averaged component’s time course. Finally, the best-fit components for the DAN, VAN, and DMN were separately selected in each group by the visual inspection, according to previous studies [9], [19], [29]. For each network component, maps of all subjects, regardless of group, were entered into random effect one sample t-test with the threshold p<0.05 (Family Wise Error (FWE) corrected) to create a sample-specific component map (See Figure S1) [30]. These maps were used as a mask for between-group analyses within the corresponding component.

#### Statistical analysis for networks

For each network, the one-sample t-test was used to determine the within-group FC pattern. The statistical threshold was set at p<0.01 corrected by False Discovery Rate (FDR) and a minimum cluster size of 20 voxels. A one-way analysis of covariance (ANCOVA) was performed to determine the differences in FC across the three groups, including age and year of education as covariates. The statistical threshold was set at corrected p<0.005 (combination of p<0.01 for single voxel and a minimum cluster size of 15 voxels), which was determined by Monte Carlo simulations using the AFNI AlphaSim program (http://afni.nih.gov/afni/docpdf/AlphaSim.pdf). The brain areas showing significantly different FC across three groups were identified as regions of interest (ROIs).

#### Correlation analysis

For each network, the mean Z value of each ROI was extracted using REST software (http://resting-fmri.sourceforge.net/). Pearson’s correlation analyses were performed to examine the relationship between altered FC strength and psychometric results in cirrhotic patients.

#### Classification method

The mean Z values of ROIs were used as indicators to distinguish MHE patients from other subjects. Linear discriminant analysis (LDA), a classification method that has been used in detecting various neurological diseases (such as Alzheimer disease/mild cognitive impairment [31] and attention deficit hyperactivity disorder [32]), was performed. The LDA with the leave-one-out method was conducted, according to the previous classification study [31], [33]. The mean Z values of ROIs from single or multiple networks entered into LDA model.

## Results

**Figure 2:**
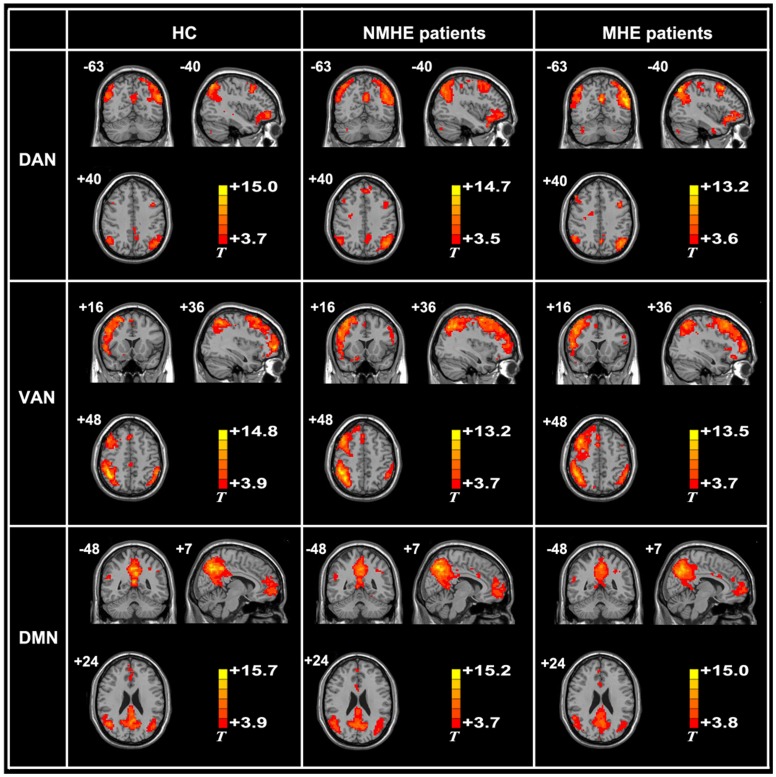
The spatial pattern of the dorsal attention network (DAN), ventral attention network (VAN), and default mode network (DMN) in healthy controls (HC), the cirrhotic patients without minimal hepatic encephalopathy (NMHE patients), and cirrhotic patients with minimal hepatic encephalopathy (MHE patients). Left side of image denotes right side of brain.

The spatial maps of the DAN, VAN and DMN were successfully obtained by Group ICA in HC, NMHE patients, and MHE patients (Figure 2). The DAN was bilateral and composed of the intraparietal sulcus and the frontal eye field; the VAN was largely right-lateralized to the temporal-parietal junction and the ventral frontal cortex; and the DMN included the precuneus/posterior cingulate cortex, medial prefrontal cortex, and bilateral inferior parietal lobule and lateral temporal lobes. The spatial patterns of these networks were consistent with previous reports [9], [29].

**Figure 3:**
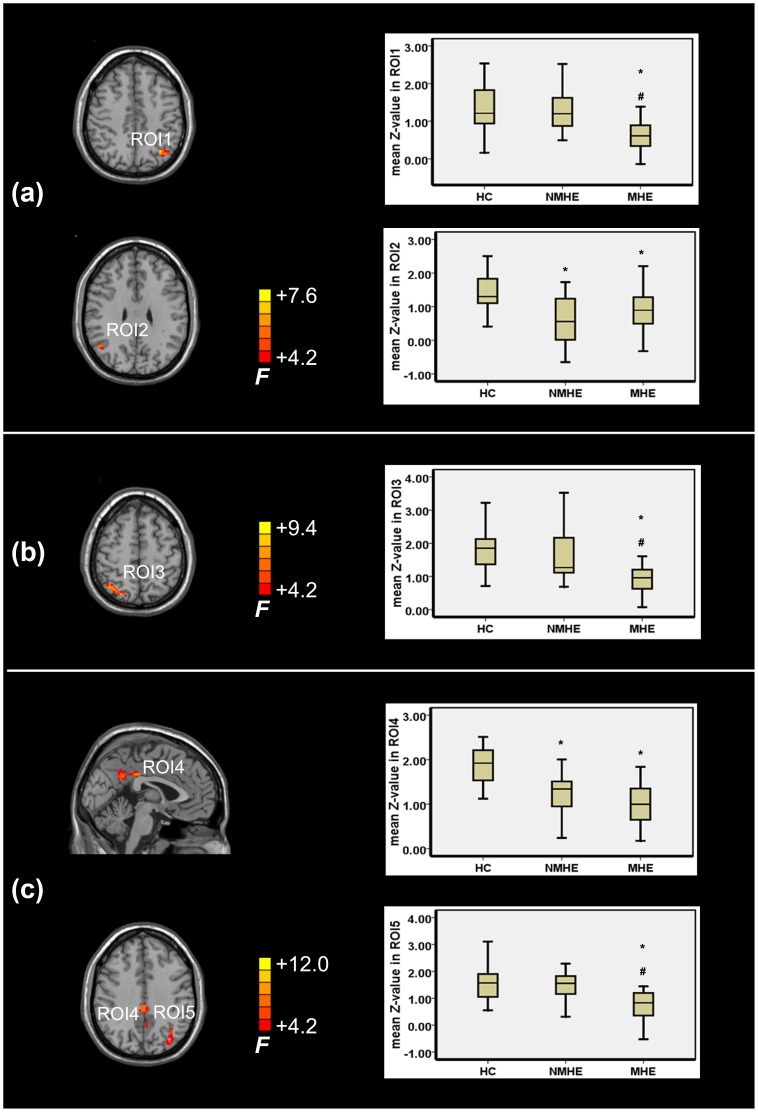
Regions showing significantly different functional connectivity across the three groups. (a) Within the dorsal attention network (DAN): ROI-1, left superior/inferior parietal lobule; ROI-2, right inferior parietal lobule. (b) Within the ventral attention network (VAN): ROI-3, right superior parietal lobule. (c) Within the default mode network (DMN): ROI-4, bilateral posterior cingulate gyrus and precuneus; ROI-5, left inferior parietal lobule. Left side of image denotes right side of brain. * indicates that cirrhotic patients showed significant decrease (p<0.05) in functional connectivity strength, compared with healthy controls (HC); while # indicates that cirrhotic patients with minimal hepatic encephalopathy (MHE) showed significant decreased (p<0.05) functional connectivity strength, compared with those without MHE (NMHE).

Figure 3 shows the result of one-way ANCOVA analysis among the three groups. Within the DAN, significant FC differences were observed in left superior/inferior parietal lobule and right inferior parietal lobule. Within the VAN, significant FC change was observed in right superior parietal lobule. Within the DMN, significant FC differences were found in bilateral posterior cingulate gyrus and precuneus, and left inferior parietal lobule. Table 2 shows detailed lists of the regions. These regions were labeled as ROI1∼ROI5 and used in sequent analyses. In addition, MHE patients showed significant FC decrease in these ROIs, compared with HCs or NMHE (Figure 3).

**Table 2:** Regions showing significantly different functional connectivity within attention-related brain networks across the three groups.

Regions	Voxels	Brodmann area	MNI coordinates			Peak *F*-value
			x	y	z	
**1) Within dorsal attention network**						
Left superior/inferior parietal lobule	21	40/39/7	−33	−63	45	7.57
Right inferior parietal lobule	16	39	48	−60	30	6.25
**2) Within ventral attention network**						
Right superior parietal lobule	48	7/40	33	−66	54	9.39
**3) Within default mode network**						
Bilateral posterior cingulate gyrus and precuneus	63	31/23/7	−6	−54	27	12.00
Left inferior parietal lobule	23	39	−36	−63	36	6.60

Table 3 shows the results of correlation analysis. Altered FC strength in attention-related networks was negatively correlated with the result of NCT-A, in which longer time to complete test indicates worse performance; while altered FC strength in attention-related networks was positively correlated with the result of DST and BDT, in which lower score indicates worse performance.

**Table 3:** Correlation between altered functional connectivity (FC) strength and psychometric results in cirrhotic patients.

Region with altered FC strength	NCT-A	DST	BDT
**1) Within dorsal attention network**			
Left superior/inferior parietal lobule	−0.56 *	0.61 *	0.46 *
Right inferior parietal lobule	0.05	−0.19	−0.06
**2) Within ventral attention network**			
Right superior parietal lobule	−0.46 *	0.46 *	0.23
**3) Within default mode network**			
Bilateral posterior cingulate gyrus and precuneus	−0.16	0.23	0.34 *
Left inferior parietal lobule	−0.40 *	0.49 *	0.46 *

NCT-A, Number Connection Test A; DST, Digit symbol test; BDT, Block design test. * indicates p value <0.05.

Single network analysis yielded relatively high classification accuracy between MHE and HC, but not between MHE and NMHE (Table 4). With FC strength of ROIs from DAN (ROI1 and ROI2 in Figure 3), VAN (ROI3 in Figure 3), and DMN (ROI4 and ROI5 in Figure 3) as indicators, LDA analysis respectively showed 75.7%, 75.7%, 86.5% classification accuracy, between MHE group and HC group; and respectively yielded 73.2%, 63.4%, 73.2% classification accuracy, between MHE group and NMHE group.

**Table 4:** Results of linear discriminant analysis between MHE patients and NMHE patients or between MHE patients and HC, with the functional connectivity strength of ROIs from single attention-related networks as indicators.

	Classification	Number of MHEsubjects (*n* = 20)	Number of HCsubjects (*n* = 17)	Classification	Number of MHEsubjects (*n* = 20)	Number of NMHEsubjects (*n* = 21)
**1) Dorsal attention network**						
Original	MHE group	17 (85.0%)	5 (29.4%)	MHE group	15 (75.0%)	5 (23.8%)
	HC group	3 (15.0%)	12 (70.6%)	NMHE group	5 (25.0%)	16 (76.2%)
Cross-validated	MHE group	16 (80.0%)	5 (29.4%)	MHE group	15 (75.0%)	6 (28.6%)
	HC group	4 (20.0%)	12 (70.6%)	NMHE group	5 (25.0%)	15 (71.4%)
**2) Ventral attention network**						
Original	MHE group	16 (80.0%)	5 (29.4%)	MHE group	15 (75.0%)	10 (47.6%)
	HC group	4 (20.0%)	12 (70.6%)	NMHE group	5 (25.0%)	11 (52.4%)
Cross-validated	MHE group	16 (80.0%)	5 (29.4%)	MHE group	15 (75.0%)	10 (47.6%)
	HC group	4 (20.0%)	12 (70.6%)	NMHE group	5 (25.0%)	11 (52.4%)
**3) Default mode network**						
Original	MHE group	18 (90.0%)	3 (17.6%)	MHE group	14 (70.0%)	5 (23.8%)
	HC group	2 (10.0%)	14 (82.4%)	NMHE group	6 (30.0%)	16 (76.2%)
Cross-validated	MHE group	18 (90.0%)	3 (17.6%)	MHE group	14 (70.0%)	5 (23.8%)
	HC group	2 (10.0%)	14 (82.4%)	NMHE group	6 (30.0%)	16 (76.2%)

Note: NMHE, the cirrhotic patients without MHE; MHE, minimal hepatic encephalopathy; HC, healthy control; ROI, regions of interest.

With FC strength of ROIs from three networks as indicators, the optimal classification results could be obtained. LDA analysis showed 94.6% classification accuracy, with 100% sensitivity and 88.2% specificity, between MHE group and HC group; and yielded 85.4% classification accuracy, with 90.0% sensitivity and 81.0% specificity, between MHE group and NMHE group (Table 5). With the FC strength of ROI1∼ROI5 (see Figure 3) as five features, the discriminate score can be calculated by the below function between MHE and HC groups: *Function* = 1.074×*F1*−0.218×*F2*+0.760×*F3*+0.309×*F4*+1.192×*F5*–3.549; while the discriminate score can be calculated by the below function between MHE and NMHE groups: *Function* = 0.708×*F1*+0.844×*F2*+1.105×*F3*+1.387×*F4*+0.682×*F5*–5.840.

**Table 5:** Results of linear discriminant analysis between MHE patients and NMHE patients or between MHE patients and HC, with the functional connectivity strength of ROIs from three attention-related networks as indicators.

	Classification	Number of MHEsubjects (*n* = 20)	Number of HCsubjects (*n* = 17)	Classification	Number of MHEsubjects (*n* = 20)	Number of NMHEsubjects (*n* = 21)
Original	MHE group	20 (100%)	0 (0%)	MHE group	18 (90.0%)	2 (9.5%)
	HC group	0 (0%)	17 (100%)	NMHE group	2 (10.0%)	19 (90.5%)
Cross-validated	MHE group	20 (100%)	2 (11.8%)	MHE group	18 (90.0%)	4 (19.0%)
	HC group	0 (0%)	15 (88.2%)	NMHE group	2 (10.0%)	17 (81.0%)

Note: NMHE, the cirrhotic patients without MHE; MHE, minimal hepatic encephalopathy; HC, healthy control; ROI, regions of interest.

## Discussion

In this study, we detected changed FC within attention-related brain networks (i.e. DAN, VAN, and DMN) in cirrhotic patients, which reflected disrupted functional integration in attention domain and may play an important role in patients’ attention deficit. Moreover, we found that these alterations can be used to classify MHE patients from NMHE patients or HC. This finding is helpful, because it indicated that attention-related network analysis based on resting-state functional MR imaging may provide a new insight into MHE detection which is important for clinicians given that prompt treatment has been demonstrated to improve MHE patients’ cognitive functions and health-related quality of life [34] and to reduce the damage MHE causes to individuals and society [35].

Our finding of disconnections in DAN, VAN, and DMN is consistent with previous reports about MHE [15], [16]. Qi and the colleagues have reported decreased FC in DAN [15]; while disruption of default-mode connectivity has been also found in MHE [14], [16]. The decreased FC strength may be associated with cerebral metabolic and structural changes. For example, the disrupted FC in the DAN and DMN has been demonstrated to correlate with increased blood ammonia level in HE patients [15]. Also, Lin and the colleagues reveal that the decreased default-mode connectivity is correlated with cerebral edema (that is associated with metabolic disturbance of ammonia) [14], [36] and impaired microstructure of white matter [37]. On the other hand, we also found a trend of more decreased FC from NMHE to MHE, suggesting the progressive impairments in attention-related networks with the disease advancement. This finding was in accordance with previous structural and functional reports about HE patients [15], [38], [39], [40], [41], [42] and further supported the concept that HE is a continuum of neurocognitive dysfunction [43].

It has been well documented that MHE leads to dysfunction in multiple attention subsystems [3], [4]. Altered FC within attention-related networks might play an important role in the attention deficit of MHE patients. Many behavioral studies reveal that MHE patients show poor performance in the selective attention-related tasks (e.g., Stroop test) and divided attention-related tasks (e.g., Number Connection Test B) [4], [6], [8], [24], [44]; while the disrupted DAN has been demonstrated to be associated with the declines in these attention domains [9], [10]. Another impaired attention domain MHE patients commonly have is the sustained attention [4], which is involved in the function of VAN [9], [10]. The disruption of DMN can also result in disturbed coordination of attention process, given that the DMN is a core network negatively modulating attention process [11], [13]. Thus, the changed FC in attention-related networks implied the decline of capabilities both in the endogenous attention orienting (“top-down”) process and the exogenous attention re-orienting (“bottom-up”) process, which may be an important neuropathological mechanism underlying attention deficit and may in turn lead to various neurocognitive impairments in consideration of the fundamental role of attention in the cognitive function. These implications above were supported by our results in correlation analyses, in which negative relationship between altered FC and NCT-A result and positive relationship between altered FC and DST/BST results, were found.

Our results showed that measuring altered FC strength is useful in classification of subjects with or without MHE and HC. MHE represents the mild stage of continuous neurocognitive dysfunction in cirrhotic patients, which can not be detected by routine clinical tests [43]. It is significant to discover new biomarker for MHE. It has been reported that impaired attention domain (e.g. anterior attention system) is sensitive to the early stage of hepatic encephalopathy [6]. On the other hand, due to the high resting metabolism [45], the regions within DMN can be particularly vulnerable to the brain metabolic disturbance in MHE. Altered FC strength in attention-related networks can be used to discriminate MHE patients from HCs, which preliminarily suggested the diagnostic potential of these indicators. Indeed, previous studies have shown that MHE can be detected by measuring attention deficit in cirrhotic patients [5], [18]. Of note, the single network analysis can not be used to adequately distinguish MHE from NMHE, due to that NMHE patients can also have subtle alteration of FC in attention-related network. An optimal discrimination result was obtained by combination of all indicators from three attention-related networks, suggesting that measuring multiple networks can more comprehensively characterize MHE condition. The improvement in discrimination power between NMHE and MHE may be derived from the fact that the three networks engage in distinct processes of attention which are functionally complementary and coordinating [9], [10], [11]. Our classification power between NMHE and MHE (90.0% sensitivity and 88.2% specificity) was similar to previous reports in which MHE was detected through measuring attention deficit as well [5], [18]. It is noted that the attention-related network analysis produced the relatively lower or equal discrimination power, as compared to several MHE diagnostic methods such as critical flicker frequency [46] and computerized inhibitory control test [24], because it only examine the one of impaired neurocognitive domains in MHE. This suggested that resting-state FC changes within attention-related networks can be the alternative diagnostic biomarker for MHE and supplementary with other existing MHE diagnostic methods.

Several limitations in this study should be acknowledged. The first one was the relatively small sample size that may limit the statistical power, although LDA with the leave-one-out method was conducted. Secondly, we confined the age and education level of subjects to increase the population homogeneity and decrease influences of these variables on classification results. Thus, whether our classification result can extend into other cirrhotic patients needs to be validated in the future. Another limitation was that this report represented a cross-sectional study. The classification result can be further validated by using a different cohort or longitudinal data. Despite these limitations, this study was the first report about classification of subjects with MHE or NMHE, and HC by measuring attention-related brain networks.

## Conclusion

The FC changes in attention-related brain networks might play an important role in the mechanisms underlying attention deficits in MHE. The resting-state attention-related brain network analysis based on the resting-state functional MR imaging can be useful in classification of subjects with MHE, NMHE, and HC, which provided a new insight into MHE detection.

## Supporting Information

Figure S1 **The masks of three attention-related networks.** DAN, dorsal attention network; VAN, ventral attention network; DMN, default mode network.
(TIF)
