# Supplementary figures and images for: Classification of Cirrhotic Patients with or without Minimal Hepatic Encephalopathy and Healthy Subjects Using Resting-State Attention-Related Network Analysis
Source: PLoS One. 2014 Mar 19;9(3):e89684. doi: 10.1371/journal.pone.0089684 (PMC3960105; doi:10.1371/journal.pone.0089684)

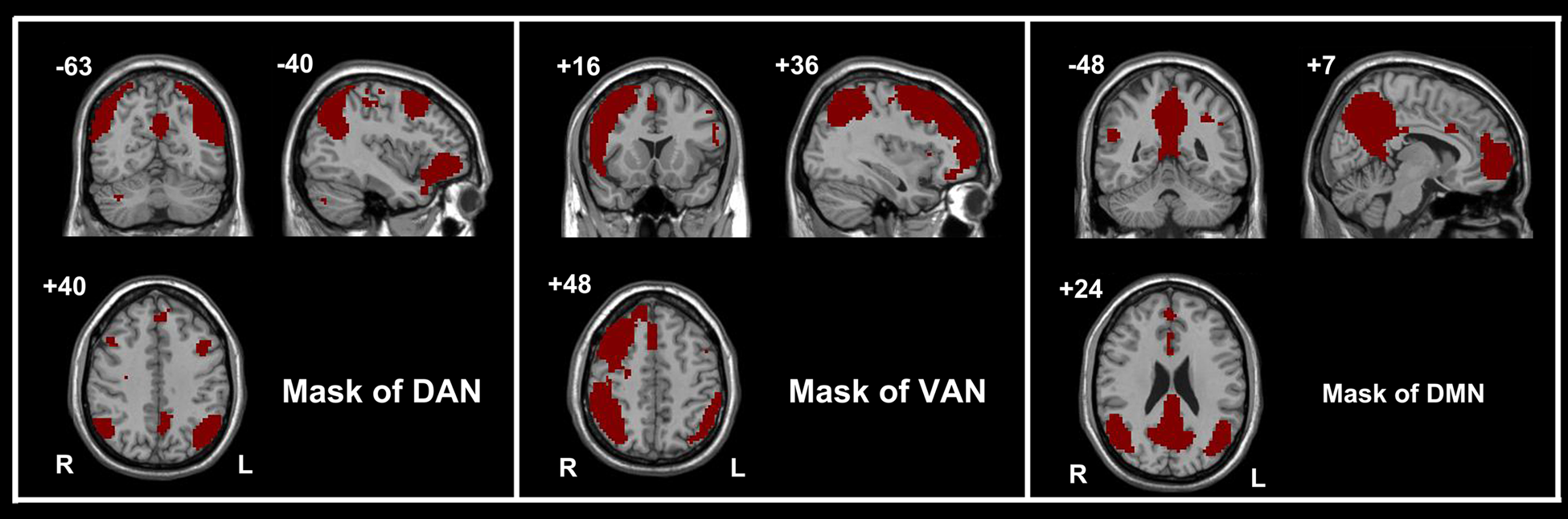

Supplement: Figure S1 — The masks of three attention-related networks. DAN, dorsal attention network; VAN, ventral attention network; DMN, default mode network. (TIF) [file pone.0089684.s001.tif]
